# Supplementary figures and images for: STING promotes NLRP3 localization in ER and facilitates NLRP3 deubiquitination to activate the inflammasome upon HSV-1 infection
Source: PLoS Pathog. 2020 Mar 18;16(3):e1008335. doi: 10.1371/journal.ppat.1008335 (PMC7080238; doi:10.1371/journal.ppat.1008335)

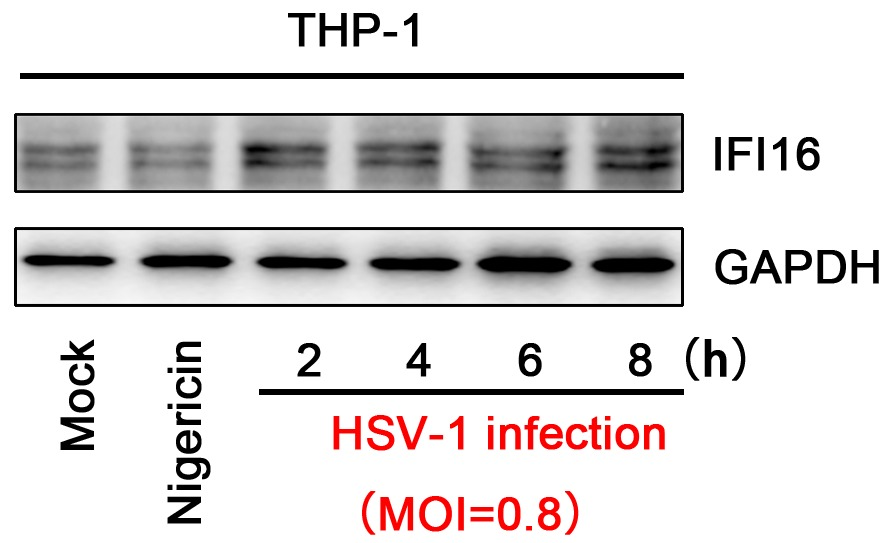

Supplement: S1 Fig — TPA-differentiated THP-1 macrophages were treated with 2 μM Nigericin for 2 h, and infected with HSV-1 at MOI = 0.8 for 2, 4, 6 and 8 h. The expression of IFI16 during HSV-1 infection was determined by Western-blot analyses. (TIFF) [file ppat.1008335.s001.tiff]

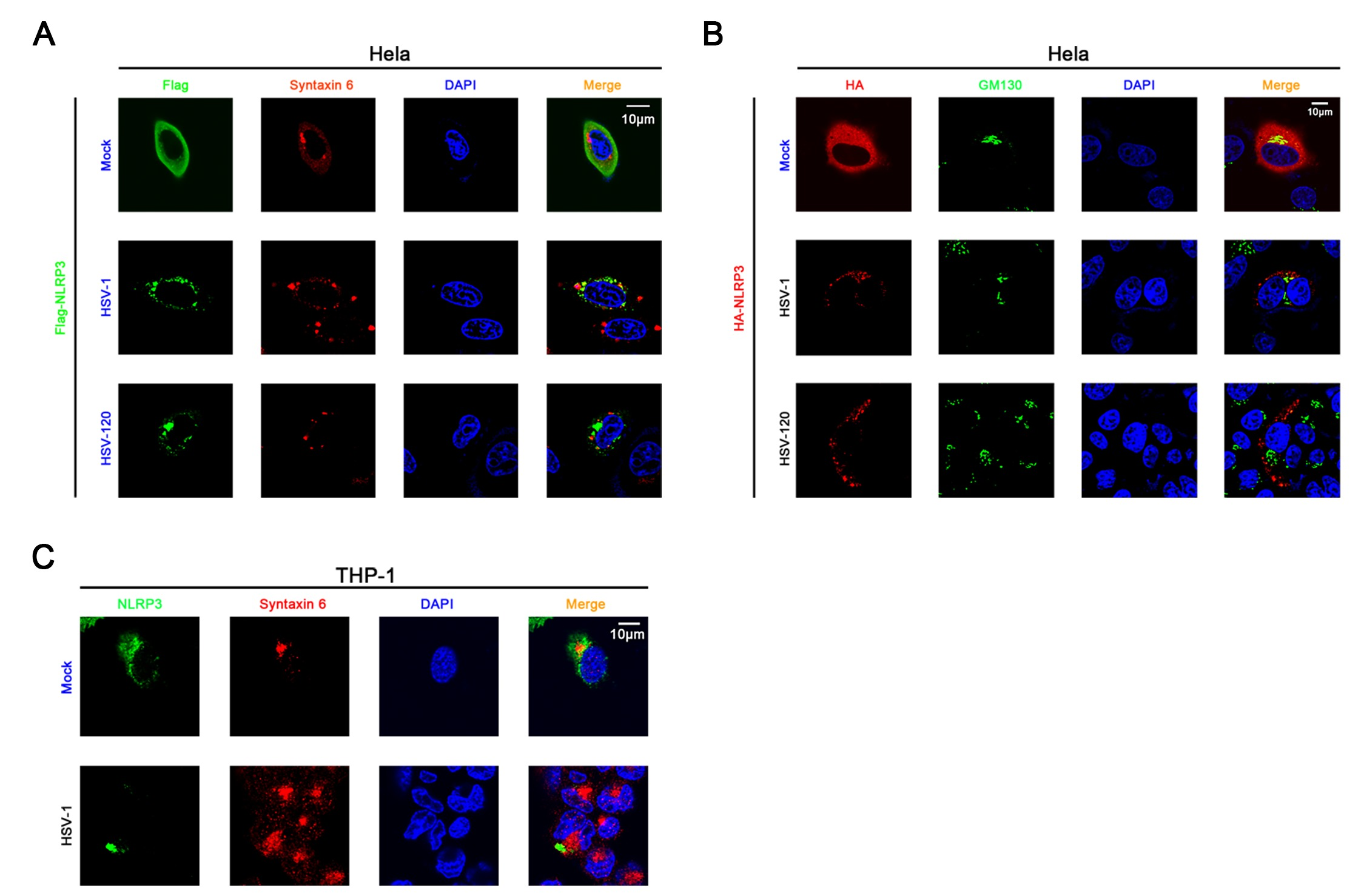

Supplement: S2 Fig — (A) Hela cells were transfected with pFlag-NLRP3 and infected with HSV-1 (MOI = 1) for 4 h or transfected with HSV120 (3 μg/ml) for 4 h. Sub-cellular localization of Flag-NLRP3 (green), Syntaxin 6 (TGN marker, red) and DAPI (blue) were examined by confocal microscopy. (B) Hela cells were transfected with pHA-NLRP3 and infected with HSV-1 (MOI = 1) for 4 h or transfected with HSV120 (3 μg/ml) for 4 h. Sub-cellular localization of HA-NLRP3 (red), GM130 (cis Golgi marker, green) and DAPI (blue) were examined by confocal microscopy. (C) TPA-differentiated THP-1 macrophages were infected with mock or HSV-1 (MOI = 1) for 4 h. Sub-cellular localization of NLRP3 (green), Syntaxin 6 (TGN marker, red) and DAPI (blue) were examined by confocal microscopy. (TIFF) [file ppat.1008335.s002.tiff]
