# Supplementary material for: STING promotes NLRP3 localization in ER and facilitates NLRP3 deubiquitination to activate the inflammasome upon HSV-1 infection
Source: PLoS Pathog. 2020 Mar 18;16(3):e1008335. doi: 10.1371/journal.ppat.1008335 (PMC7080238; doi:10.1371/journal.ppat.1008335)
Supplement: Attachment — Submitted filename: PLoS Pathog Responses to the Reviews 20191204.docx [file ppat.1008335.s003.docx]

**Responses to the Reviews**

**Part I – Summary**

Please use this section to discuss strengths/weaknesses of study, novelty/significance, general execution and scholarship.

**Reviewer #1:** In this paper, Wu and coworkers suggested the requirement of cGAS-STING pathway of DNA sensing for activation of NLRP3 inflammasome during HSV-1 infection. It was previously hypothesized that HSV-1 induces NLRP3 inflammasome through an unknown mechanism. This work provides one possible explanation and some new insight on how host activates NLRP3 inflammasome. However, while some results are interesting, others are redundant and confirmatory. In addition, several key questions remain unanswered. Some parts are disconnected. There are major concerns that have to be addressed. Specific comments are as follows.

**Authors’ Response:** Thank you very much for the comments. We have addressed your questions in the revision.

**Reviewer #2:** Manuscript: Wenbiao Wang et al: STING recruits NLRP3 to the ER and deubiquitinates NLRP3 to activate the inflammasome upon DNA virus infection

Background: Inflammasome induction and type I The DNA Inflammasome are the first line of defense in eukaryotic cells against viruses etc. The presence of cytosolic DNA is recognized by several pattern receptors- the cGAS-STING axis triggers type I interferon response and the NLRP3 and AIM2 axis triggers the inflammasome axis. NLRP3 is activated by a broad range of pathogen-derived and endogenous agents including mitochondrial DNA etc.

Recent studies reveal the complexity of these stimulation, cell type differences, and the cross-interaction and stimulation among the components. For example:

A. Feb 2017 Nature communications: Studies demonstrate that “ IFI16 and cGAS cooperate in the activation of STING during DNA sensing in human keratinocytes”
B. Cell 2017 studies demonstrate that “DNA in Human Myeloid Cells Is Initiated by a STING-Cell Death Program Upstream of NLRP3” Cell 2017 studies show that cell type-specificities and differences between primary monocytes and THP-1 cells.

1) In human monocytic cell line THP-1 and murine BMDMs (bone marrow derived macrophages), AIM2 mediates the cytosolic DNA recognition, inflammasome activation and IL-1b secretion, and not by NLRP3 in THP-1 cells, and pharmacologic inhibition of NLRP3 had no impact on DNA-mediated inflammasome activation in these cells

2) In contrast, NLRP3 is involved in the inflammasome induction after cytosolic DNA recognition in human monocytes from peripheral blood.

C. J Expt medicine 2017 paper show that cGAMP has a noncanonical function in inflammasome activation in human and mouse cells.

In BMDMs, cGAMP activates the inflammasome through an AIM2, NLRP3, ASC, and caspase-1 dependent process, requiring differential requirements for STING. cGAMP induction of IFN-I preceded the inflammasome activation, and both cGAS/cGAMP amplify both inflammasome and IFN-I responses to control murine cytomegalovirus in mice.

D. HSV-1 tegument protein VP22 of HSV-1 has been shown to inhibit AIM2 inflammasome induction.

E. Human fibroblast cell infection induces the NLRP3-ASC interaction o-4 h infection but not later.

**Authors’ Response:** Thank you very much for the comments.

**Major:**

This manuscript follows some of these papers to determine the induction of NLRP3 inflammasome induction by HSV-1.

While some findings are very interesting, this manuscript is written in a brief format including abstract, introduction, results and discussion section. A difficult read and several experimental details are lacking, and hence difficult to follow, lacks appropriate controls and very selective. See the comments under individual figures.

**Authors’ Response:** Thank you for the comments. Our results reveal a distinct mechanism underlying STING-mediated NLRP3 inflammasome activation, and demonstrate for the first time that STING binds to NLRP3 and promotes the inflammasome activation through two approaches. First, STING binds to and improves NLRP3 localization in ER to promote the formation of the NLRP3 inflammasome. Second, STING interacts with NLRP3 and decreases K48- and K63-linked polyubiquitination of NLRP3 to derepress the activation of the NLRP3 inflammasome.

We have added the experimental details and control description in the revised manuscript.

The overall conclusion Fig 9- Role of cGAS in viral DNA recognition and activation of STING is not shown in any of the figures given.

**Authors’ Response:** Thank you for the comment.

As you suggested, we constructed and analyzed THP-1 cell lines stably expressing negative control shRNA (sh-NC) and shRNAs to IFI16 and cGAS (sh-IFI16 and sh-cGAS). The new results in the revised manuscript revealed that IL-1β secretion (Revised Fig 4A) as well as IL-1β (p17) cleavage and Casp-1 (p20 and p22) maturation (Revised Fig 4B) induced by HSV-1, not Nigericin, were attenuated by sh-IFI16 and sh-cGAS.

Title: STING recruits NLRP3 to the ER and deubiquitinates NLRP3 to activate the inflammasome upon DNA virus infection “upon DNA virus infection” – They have tested only HSV-1. If they have also tested cytoplasmic replicating vaccinia virus as a control – it will be valid- as vaccinia virus has been shown to stimulate AIM2 in human cells.

**Authors’ Response:** Thank you for your comment. As you indicated, we have changed the original title “STING recruits NLRP3 to the ER and deubiquitinates NLRP3 to activate the inflammasome upon DNA virus infection” to “STING promotes NLRP3 localization in ER and facilitates NLRP3 deubiquitination to activate the inflammasome upon HSV-1 infection” in the revised manuscript.

**Reviewer #3:** This manuscript describes that DNA virus activates NLRP3 inflammasome through STING. It shows NLRP3 inflammasome could be activated by viral DNAs through STING pathway in addition to the well-known ROS and other PAMPs, STING-mediated NLRP3 inflammasome activation is mainly through recruiting NLRP3 to the ER and removing K48- and K63-linked polyubiquitination of NLRP3. This manuscript is quite interesting that it extends the range of NLRP3 activation and offers a novel perspective of STING’s function in unification of innate immunity and inflammasome.

**Authors’ Response:** Thank you very much for your comments.

**Reviewer #4:** In this study, the authors reveal a distinct function of STING of regulating NLRP3 dependent inflammasome activation in multiple cells/cell lines upon HSV-1 infection or cytosolic DNA stimulation. Mechanistically, the authors provide strong evidence that STING binds to NLRP3 in ER and therefore stabilizing the inflammasome formation. Furthermore, they also showed that STING overexpression is associated with attenuation of both K48- and K63-linked polyubiquitination of NLRP3, thereby promoting the inflammasome activation. This is a very interesting and solid study with decent amounts of supporting data. I would like to recommend that the authors perform some extra experiments, discuss more about the mechanism and make more appropriate conclusions.

**Authors’ Response:** Thank you very much for your comments. As you advised, we have performed extra experiments, discussed more about the mechanism and made more appropriate conclusions in the revised manuscript.

**Part II – Major Issues: Key Experiments Required for Acceptance**

Please use this section to detail the key new experiments or modifications of existing experiments that should be absolutely required to validate study conclusions.

Generally, there should be no more than 3 such required experiments or major modifications for a "Major Revision" recommendation. If more than 3 experiments are necessary to validate the study conclusions, then you are encouraged to recommend "Reject".

**Reviewer #1:**

1) Many key questions that should be addressed and can be easily addressed were left untouched. These include the following and at least some should be experimentally addressed:

i) The role of cGAS: It was mentioned in the abstract that cGAS-STING-NLRP3 pathway is essential for host defense against DNA virus infection. However, no experiment was done to build the link with cGAS.

ii) The role of cGAMP: Is STING activated by cGAMP or directly by DNA or anything else?

iii) The identity of the deubiquitinase recruited.

iv) Does STING per se have deubiquitinase activity towards NLRP3?

**Authors’ Response:** Thank you for your comments.

i) As you suggested, we have carried out additional experiments to build the link with cGAS in the revised manuscript (Revised Fig. 4A and B).

ii) It has been demonstrated that DNA transfection or DNA virus infection of mammalian cells trigger the production of cGAMP that subsequently binds to STING, leading to the activation of IRF3 and induction of interferon-β ([Science](https://www.ncbi.nlm.nih.gov/pubmed/?term=PMID%3A+23258412) 2013 Feb 15; 339(6121): 826-30. PMID: 23258412).

iii) and iv) As we known, STING has no DUB function. It was reported that EIF3S5 ([Nat Immunol](https://www.ncbi.nlm.nih.gov/pubmed/?term=PMID%3A+27428826) 2016 Sep; 17(9): 1057-66. PMID: 27428826) and USP20 ([Cell Res](https://www.ncbi.nlm.nih.gov/pubmed/?term=PMID%3A+27801882) 2016 Dec; 26(12): 1302-1319. PMID: 27801882) were recruited for the deubiquitination of STING by other proteins. However, the DUB for deubiquitination of NLRP3 mediated by STING is unknown. We thus have changed “STING interacts with NLRP3 and removes K48- and K63-linked polyubiquitination of NLRP3” to “STING interacts with NLRP3 and attenuates K48- and K63-linked polyubiquitination of NLRP3” in the revised manuscript.

2) Why were LPS and nigericin used to prime cells (Fig. 3, 4) if HSV-1 alone can sufficiently activate both signals for inflammasome activation (Fig. 7, 8).

**Authors’ Response:** Thank you for your comments. Primary MEFs were primed with LPS (1 µg/ml) for 6 h. The nigericin was used as a positive control. The activation of IL-1β secretion is regulated by two pathways in response to pathogen infections: transcription of pro-IL-1β and procession by Caspase-1 ([Cell](https://www.ncbi.nlm.nih.gov/pubmed/?term=PMID%3A+20303873) 2010 Mar 19; 140(6): 821-32. PMID:20303873). The transcription of pro-IL-1β is regulated by NF-κB pathway. Bacterial lipopolysaccharide (LPS) was used as an activator of the NF-κB pathway ([Cell](https://www.ncbi.nlm.nih.gov/pubmed/?term=PMID%3A+3096580) 1986 Dec 26; 47(6): 921-8. PMID: 3096580). Nigericin is a bacterial toxin that activates NLRP3 by causing potassium efflux in a pannexin-1-dependent pathway ([Nature](https://www.ncbi.nlm.nih.gov/pubmed/?term=PMID%3A+19339971) 2009 May 21; 459(7245): 433-6. PMID: 19339971). In TPA-differentiated THP-1 macrophages, HSV-1 alone can activate both signals for inflammasome activation, but not in the primary MEFs.

**Reviewer #2:**

**Fig. 1.** HEK293T cells were used (A-L). These cells lack several innate response proteins most notable cGAS, IFI16 etc. (Proc Natl Acad Sci U S A. 2015 Apr 7; 112(14): E1773–E1781.

**Authors’ Response:** Thank you for your comments. We only used HEK293T cells for the analyses of the NLRP3/STING interaction, not for the determination of NLRP3 and STING functions. The functional analyses of NLRP3 and STING were determined in other cell types, including in TPA-differentiated human leukemic monocyte (THP-1) macrophages and primary mouse embryo fibroblasts (MEFs), in the original manuscript. Additionally, we have used THP-1 cell lines stably expressing negative control shRNA (sh-NC) and shRNAs (sh-IFI16, sh-cGAS, and sh-STING) in the revised manuscript (Revised Fig. 4).

**- Fig 9**- Steady state – STING and NLRP3 do not interact- yet they show over expression of these molecules in 293T and HeLa they interact? How?

- Appropriate controls are lacking.

- Why STING and NLRP3 interacts in the absence of cGAS or cGAMP and any external stimulation? How inflammasome is induced in the absence of recognition of DNA? Or other stimulus? Not explained and very difficult to conclude without the experimental details

- Show cGAS?

Line 116-117 – Explain the system.

**Authors’ Response:** Thank you for your comments.

- Yes, we showed that STING and NLRP3 do not interact in the steady state in macrophage. However, in TPA-differentiated human leukemic monocyte (THP-1) macrophages, HSV-1 infection facilitates the interaction of endogenous NLRP3 with STING and promotes the interaction of endogenous STING with NLRP3 (Fig 2A, B). Moreover, in LPS-primed primary mouse embryo fibroblasts (MEFs), HSV-1 infection and HSV120 transfection facilitate endogenous STING/NLRP3 interaction (Fig 2H).

**-** Appropriate controls have been added to all experiments in the revision.

**-** HEK293T cells were co-transfected with pFlag-STING and pHA-NLRP3. This overexpression system indicates that NLRP3 may interact with STING. TPA-differentiated human leukemic monocyte (THP-1) macrophages, HSV-1 infection facilitated endogenous NLRP3-STING interaction (Fig 2A, B). It has been demonstrated that overexpression system can induce the NLRP3 inflammasome activation without stimulation, such as the four-plasmid system in HEK293T ([J Virol](https://www.ncbi.nlm.nih.gov/pubmed/?term=PMID%3A+21994456) 2011 Dec; 85(24): 13019-26. PMID: 21994456; [Cell Rep](https://www.ncbi.nlm.nih.gov/pubmed/?term=PMID%3A+26119741) 2015 Jul 7; 12(1):42-48. PMID: 26119741).

**-** As you suggested, we have carried out additional experiments to build the link with cGAS in the revised manuscript (Revised Fig. 4A and B). It has been demonstrated that DNA transfection or DNA virus infection of mammalian cells trigger the production of cGAMP that subsequently binds to STING, leading to the activation of IRF3 and induction of interferon-β ([Science](https://www.ncbi.nlm.nih.gov/pubmed/?term=PMID%3A+23258412) 2013 Feb 15; 339(6121): 826-30. PMID: 23258412).

- The pro-Casp-1 activation and pro-IL-1β cleavage cell system (NACI) has been explained in the revised manuscript and well described previously ([PLoS Pathog.](https://www.ncbi.nlm.nih.gov/pubmed/28060938) 2017 Jan 6;13(1):e1006123. PMID: 28060938). In this system, HEK293T cells were co-transfected with plasmids expressing the components of the NLRP3 inflammasome, NLRP3, ASC, and pro-Casp1, and pro-IL-1β.

**Fig 2:**

-Appropriate controls lacking.

- Infection procedures are not clear. What is 2 h/ is it 2 h incubation with virus, wash and then 2 and 4 h infection?

- What is Mock? Not described.

- Lines 434-436: HSV-1 was collected by freezing and thawing infected Vero cells- Hence, this is not a purified enveloped virus preparation. This preparation with contain enveloped viruses, viral DNA, host nuclear and mitochondrial DNA, viral capsids with and without daughter DNA, cell debris. All these materials added to the cell can be taken up by endocytosis and can induce the stimulus.

- TPA induced THP1 cells were used for HSV-1 infection (2-4 h) - AIM2 has been shown to induce the inflammasome after recognition of the cytoplasmic DNA in these cells – see the background above.

- No AIM2 controls.

**Authors’ Response:** Thank you for the comments.

- The infection time was the incubation time with the virus HSV-1.

- The mock-infected was prepared at the same Vero cells without HSV-1 infection, but others are the same procedure. As the virus infection time is too short, the infection procedures are 2 h and 4 h infection without wash.

- Vero cells were inoculated with HSV-1 at MOI=0.03 for 36 h. The infected cells were collected, HSV-1 stocks were propagated after three times of freezing and thawing, and the viral titers were determined by plaque assay in 12-well plates in Vero cells. TPA-differentiated THP-1 macrophages were mock-infected or infected with HSV-1 at MOI=1 for 4 h (Revised Fig 2A) or 2 and 4 h (Revised Fig 2B). HEK293T cells (Revised Fig 2C) and Hela cells (Revised Fig 2D) were co-transfected with pFlag-STING and pHA-NLRP3, and then infected with HSV-1 (MOI=1) for 2–4 h.

- In parallel, Vero cells were inoculated with culture medium for 36 h. The cells were lysates after three times of freezing and thawing and used as negative control (Mock). TPA-differentiated THP-1 macrophages were mock-infected (inoculated with negative control cell lysates) for 4 h (Revised Fig 2A) or 2 and 4 h (Revised Fig 2B). HEK293T cells (Revised Fig 2C) and Hela cells (Revised Fig 2D) were co-transfected with pFlag-STING and pHA-NLRP3, and then mock-infected (inoculated with negative control cell lysates) for 2–4 h.

**-** To avoid the concerns that you have raised, we used the mock-infection as negative control to exclude the possibility that host nuclear DNA, mitochondrial DNA, cell debris added to the cell can be taken up by endocytosis and can induce the stimulus.

- AIM2 has been shown to induce the inflammasome after recognition of the cytoplasmic DNA, but not HSV-1. Previous studies have demonstrated that maturation and release of IL-1β in response to HSV-1 was normal in cells of Aim2^-/-^ mice in contrast to vaccinia virus and mouse cytomegalovirus ([Nat Immunol.](https://www.ncbi.nlm.nih.gov/pubmed/?term=PMID%3A+20351692) 2010 May; 11(5): 395-402. PMID: 20351692), and AIM2 isn’t essential for HSV-1-induced inflammasome activation ([Cell Host Microbe.](https://www.ncbi.nlm.nih.gov/pubmed/?term=PMID%3A+29447697) 2018 Feb 14; 23(2): 254-265. PMID: 29447697).

- As you suggested, we have added AIM2 control in the revised manuscript by constructing and analyzing THP-1 cell lines stably expressing negative control shRNA (sh-NC) and shAIM2. The results showed that IL-1β secretion (Revised Fig 4D) as well as IL-1β (p17) cleavage and Casp-1 (p20 and p22) maturation (Revised Fig 4E) induced by poly dA:dT were attenuated by sh-AIM2, however, IL-1β secretion (Revised Fig 4D) as well as IL-1β (p17) cleavage and Casp-1 (p20 and p22) maturation (Revised Fig 4E) induced by HSV-1 infection were unaffected by sh-AIM2.

**- Fig 2A.** Fig 1 shows interaction of NLRP3 and STING - here no endogenous interaction - explain. But there is interaction of transfected DNA- C and D – lanes 2

- HSV-1 is a nuclear replicating virus and IFI16-inflammasome and interferon responses have been shown to be stimulated- Show this here as controls.

- What about cGAS and cGAMP production in the endogenous infection conditions?

**Authors’ Response:** Thank you for your comments.

- We evaluated the effects of HSV-1 infection and HSV120 transfection, a biotinylated dsDNA representing the genomes of HSV-1 that efficiently induces STING-dependent type I IFN production as reported previously, on the STING-NLRP3 interaction. In TPA-differentiated human leukemic monocyte (THP-1) macrophages, HSV-1 infection facilitated endogenous NLRP3-STING interaction and promoted endogenous STING-NLRP3 interaction (Fig 2A, B). In HEK293T cells and HeLa cells (Fig 2D), the STING-NLRP3 interaction was enhanced upon HSV-1 infection (Fig 2C, D).

- It is well known both IFI16 ([Nat Immunol](https://www.ncbi.nlm.nih.gov/pubmed/?term=PMID%3A+20890285) 2010 Nov; 11(11):997-1004. PMID: 20890285) and cGAS ([Science](https://www.ncbi.nlm.nih.gov/pubmed/?term=PMID%3A+23989956) 2013 Sep 20; 341(6152): 1390-4. PMID: 23989956) are innate DNA sensors mediating interferon-β-induction after HSV-1 infection. Both the interferon response and the inflammasome activation are important in innate immune system and the relationship is complicate.

- After HSV-1 infection, the cGAS binds to DNA independently of its sequence, which leads to the activation of cGAS to catalyze the synthesis of a unique cGAMP isomer that contains both 2’–5’ and 3’–5’ phosphodiester linkages. This molecule, termed 2’3’cGAMP, functions as a second messenger that binds and activates the adaptor protein STING. STING then activates the protein kinases IKK and TBK1, which in turn activate the transcription factors NF-κB and IRF3 to induce interferons and cytokines in the revised manuscript ([Science](https://www.ncbi.nlm.nih.gov/pubmed/?term=PMID%3A+23989956) 2013 Sep 20; 341(6152): 1390-4. PMID: 23989956).

**- Fig 2E:** Control plasmid transfection?

- So why transfection of HSV120 plasmid is not inducing AIM2 inflammasome? This lacks VP22. Hence AIM2 inflammasome will be induced.

This DNA has to enter the nucleus in order to express the viral genes (as shown in the later figures) and hence must be inducing IFI16-cGAS interferon response and IFI16-inflammasome. Hence, the results as shown are very surprising.

**-** We have used the control plasmid transfection and added the description in the revised manuscript. THP-1 cells were transfected with HSV120 by Lipo2000 and used the same Lipo2000 as the control.

**-** AIM2 has been shown to induce the inflammasome after recognition of the cytoplasmic DNA in the cells, but not HSV-1. Previous studies have demonstrated that maturation and release of IL-1β in response to HSV-1 was normal in cells of Aim2^-/-^ mice in contrast to vaccinia virus and mouse cytomegalovirus ([Nat Immunol.](https://www.ncbi.nlm.nih.gov/pubmed/?term=PMID%3A+20351692) 2010 May; 11(5): 395-402. PMID: 20351692), and AIM2 isn’t essential for HSV-1-induced inflammasome activation ([Cell Host Microbe.](https://www.ncbi.nlm.nih.gov/pubmed/?term=PMID%3A+29447697) 2018 Feb 14; 23(2): 254-265. PMID: 29447697). As you suggested, we have added AIM2 control in the revised manuscript by constructing and analyzing THP-1 cell lines stably expressing negative control shRNA (sh-NC) and shAIM2. The results showed that IL-1β secretion (Revised Fig 4D) as well as IL-1β (p17) cleavage and Casp-1 (p20 and p22) maturation (Revised Fig 4E) induced by poly dA:dT were attenuated by sh-AIM2, however, IL-1β secretion (Revised Fig 4D) as well as IL-1β (p17) cleavage and Casp-1 (p20 and p22) maturation (Revised Fig 4E) induced by HSV-1 infection were unaffected by sh-AIM2. HSV120 representing the genomes of HSV-1 is DNA oligonucleotides, not plasmid ([Cell Host Microbe.](https://www.ncbi.nlm.nih.gov/pubmed/?term=PMID%3A+25299331) 2014 Oct 8;16(4):450-61. PMID: 25299331). The sequence is 5’-AGACGGTATATTTTTGCGTTATCACTGTCCCGGATTGGACACGGTCTTGTGGGATAGGCATGCCCAGAAGGCATATTGGGTTAACCCCTTTTTATTTGTGGCGGGTTTTTTGGAGGACTT-3’. We have added the material in the revised manuscript.

**Fig. 3:**

- HSV-1 infection also induces NLRP3 and IFI16 inflammasome at early time of infection (up to 4h) and IFI16 is degraded after 2-4 h in the fibroblasts (Ref 24).

To differentiate the responses that they observed in THP-1 cells, they should show the fate of IFI16.

- Line 165: “Induced upon DNA virus infection and cytosolic DNA stimulation. - Not correct statement. Vaccinia virus control should be included.

**Authors’ Response:** Thank you for your comments.

- As you suggested, we have added the fate of IFI16 in the revised manuscript. The expression of IFI16 was increased after HSV-1 infection (Revised S1 Fig).

**-** We have changes “Induced upon DNA virus infection and cytosolic DNA stimulation.” to “Induced upon HSV-1 infection” in the revised manuscript.

**Fig.4.**

Need to show time kinetics of HSV-1 infection induced inflammasome activation – ie Il1 beta and caspase 1 cleavage. in THP1 cells and mouse cells.

- Controls for ShRNA? Show AIM2 KD.

- Needs AIM2 control in Fig L, M and N

**Authors’ Response:** Thank you for your comments.

- AIM2 has been shown to induce the inflammasome after recognition of the cytoplasmic DNA in the cells, but not HSV-1. Previous studies have demonstrated that maturation and release of IL-1β in response to HSV-1 was normal in cells of Aim2^-/-^ mice in contrast to vaccinia virus and mouse cytomegalovirus ([Nat Immunol.](https://www.ncbi.nlm.nih.gov/pubmed/?term=PMID%3A+20351692) 2010 May; 11(5): 395-402. PMID: 20351692), and AIM2 isn’t essential for HSV-1-induced inflammasome activation ([Cell Host Microbe.](https://www.ncbi.nlm.nih.gov/pubmed/?term=PMID%3A+29447697) 2018 Feb 14; 23(2): 254-265. PMID: 29447697). As you suggested, we have added AIM2 control in the revised manuscript by constructing and analyzing THP-1 cell lines stably expressing negative control shRNA (sh-NC) and shAIM2. The results showed that IL-1β secretion (Revised Fig 4D) as well as IL-1β (p17) cleavage and Casp-1 (p20 and p22) maturation (Revised Fig 4E) induced by poly dA:dT were attenuated by sh-AIM2, however, IL-1β secretion (Revised Fig 4D) as well as IL-1β (p17) cleavage and Casp-1 (p20 and p22) maturation (Revised Fig 4E) induced by HSV-1 infection were unaffected by sh-AIM2. HSV120 representing the genomes of HSV-1 is DNA oligonucleotides, not plasmid ([Cell Host Microbe.](https://www.ncbi.nlm.nih.gov/pubmed/?term=PMID%3A+25299331) 2014 Oct 8;16(4):450-61. PMID: 25299331).

**Fig. 5:**

- Show AIM2 and IFI16 controls.

- STING and NLRP3 interactions? Does it require cGAS- Not clear and need to be shown.

- Show the endogenous levels of the molecules in the Hela cells.

- Since HSV120 is biotinlated DNA- should show the colocalization of NLRP3 and cGAS

- L: Primary MEFs stably expressing sh-STNG- this is surprising- Methods?

- STING Doesn’t appear to be efficiently reduced.

- LPS primed and infected? Why? Not clear.
**Authors’ Response:** Thank you for your comments.

- We have added the AIM2 and IFI16 as controls in Fig 5G in the revised manuscript.

**-** cGAS isn’t required for the interaction between STING and NLRP3 based on the results in HEK293T, which lack several innate response proteins most notable cGAS, IFI16 etc. However, in THP-1 cells, cGAS is essential for HSV-1 infection recognition and induced the STING activation. In addition, as you suggested, we have constructed and analyzed THP-1 cell lines stably expressing negative control shRNA (sh-NC) and shAIM2. The results showed that IL-1β secretion (Revised Fig 4D) as well as IL-1β (p17) cleavage and Casp-1 (p20 and p22) maturation (Revised Fig 4E) induced by poly dA:dT were attenuated by sh-AIM2, however, IL-1β secretion (Revised Fig 4D) as well as IL-1β (p17) cleavage and Casp-1 (p20 and p22) maturation (Revised Fig 4E) induced by HSV-1 infection were unaffected by sh-AIM2.

- As you suggested, we have showed the endogenous levels of STING in the Hela cells in the revised manuscript.

- Sorry for the confusion. HSV120 was a DNA oligonucleotide, but not a biotinlated DNA ([Cell Host Microbe.](https://www.ncbi.nlm.nih.gov/pubmed/?term=PMID%3A+25299331) 2014 Oct 8;16(4):450-61. PMID: 25299331). We have added the sequence in the revised manuscript: 5’-AGACGGTATATTTTTGCGTTATCACTGTCCCGGATTGGACACGGTCTTGTGGGATAGGCATGCCCAGAAGGCATATTGGGTTAACCCCTTTTTATTTGTGGCGGGTTTTTTGGAGGACTT-3’.

**-** We used the lentivirus to construct the stably expressing sh-STING system that was mentioned in the Methods. As your suggestion, we have repeated the experiments in the revised manuscript (Revised Fig. 5M). The Primary MEFs were primed with LPS (1 µg/ml) for 6 h and then infected with HSV-1 or treated with ATP. As we know, activation of NLRP3 inflammasome is regulated by two pathways: one leads to the activation of NF-κB that subsequently induces the production of pro-IL-1β, NLRP3, and ASC, and the other is initiated by the assembly of inflammasome, resulting in the activation of Casp-1 and the maturation of IL-1β. Without the LPS primed, the primary cells, such as BMDM, BMDC, or MEFs, isn’t response for the activation of NLRP3 inflammasome.

**Fig. 7:**

- What is the effect of STING KD on type I interferon response and HSV-1 replication in these cells?

**Line 315:** “This study reveals a distinct mechanism by which the cGAS-STING-NLRP3 pathway promotes the NLRP3 inflammasome activation and IL-1β secretion upon DNA virus infection and cytosolic DNA stimulation”

**Authors’ Response:** Thank you for your comments.

- HSV-1 ICP27 mRNA was detected in HSV-1 infection cells. HSV-1 replication increased in the STING KD cells. Both Interferon responses and inflammasome activation are important in innate immune system and the relationship is complicate. We only focused on NLRP3 inflammasome activation in this manuscript.

**-** We have changed “This study reveals a distinct mechanism by which the cGAS-STING-NLRP3 pathway promotes the NLRP3 inflammasome activation and IL-1β secretion upon DNA virus infection and cytosolic DNA stimulation” to “This study reveals a distinct mechanism by which the cGAS-STING-NLRP3 pathway promotes the NLRP3 inflammasome activation and IL-1β secretion upon HSV-1 infection and cytosolic DNA stimulation” in the revised manuscript.

**Fig. 9:** Role of cGAS in viral DNA recognition and activation of STING is not shown in any of the figures given.

**Authors’ Response:** Thank you for your comments. As you suggested, we constructed and analyzed THP-1 cell lines stably expressing negative control shRNA (sh-NC) and shRNAs to IFI16 and cGAS (sh-IFI16 and sh-cGAS). The new results in the revised manuscript revealed that IL-1β secretion (Revised Fig 4A) as well as IL-1β (p17) cleavage and Casp-1 (p20 and p22) maturation (Revised Fig 4B) induced by HSV-1, not Nigericin, were attenuated by sh-IFI16 and sh-cGAS.

**Reviewer #3:** However, although potentially important and meaningful, there are several points need to be improved greatly, as outlined below, to make the conclusion more convincing and clear.

**Major points:**

**1. In Figure 1,** the author proved that NLRP3 interacts with STING, and NAD and LRR in NLRP3 play the main role, and TM2 and TM5 in STING are essential. Could the author map the smaller domains or the key residues for their interaction?

**Authors’ Response:** Thank you for the comment. We should ensure the structural integrity of domains, and thus we can’t map the smaller domains or the key residues for their interaction.

**2. In Figure 1H,** the expression of truncated proteins is not consistent, especially STING (211-379aa), thus the conclusion that TM5 of STING interacts with NLRP3 is not convincing. Furthermore, the plasmid expressing only TM5 or TM5-deleted STING plasmid should be added in this figure.

**Authors’ Response:** Thank you for the comment. As suggested, we have added the TM5-deleted STING in the revised manuscript.

**3. In Figure 1I,** TM2 was claimed to promote IL-1β secretion. To be more convincing, this figure needs more mutant STING plasmids such as TM2, TM2-deleted, TM2-TM5 and TM2-TM5-deleted constructs.
**Authors’ Response:** Thank you for the comment. As you recommended, we have added TM2, TM2-deleted, TM2-TM5 and TM2-TM5-deleted constructs in the revised manuscript.

**4.** HSV as an AIM2 inflammasome stimuli is well known for a long time. In Figure 4A, 4C, IL-1β secretion could hardly be recognized as the result of HSV stimulation. Further, it is VP22 but not intact HSV virion inhibits AIM2-dependent inflammasome according to Johnson’s paper, so it may be important to perform this experiment in AIM2 knock out THP-1 cells to identify if IL-1β would be still expressed in the same pattern as in THP-1 WT cells.

**Authors’ Response:** Thank you for the comment. AIM2 has been shown to induce the inflammasome after recognition of the cytoplasmic DNA in the cells, but not HSV-1. Previous studies have demonstrated that maturation and release of IL-1β in response to HSV-1 was normal in cells of Aim2^-/-^ mice in contrast to vaccinia virus and mouse cytomegalovirus ([Nat Immunol.](https://www.ncbi.nlm.nih.gov/pubmed/?term=PMID%3A+20351692) 2010 May; 11(5): 395-402. PMID: 20351692), and AIM2 isn’t essential for HSV-1-induced inflammasome activation ([Cell Host Microbe.](https://www.ncbi.nlm.nih.gov/pubmed/?term=PMID%3A+29447697) 2018 Feb 14; 23(2): 254-265. PMID: 29447697). As you suggested, we have added AIM2 control in the revised manuscript by constructing and analyzing THP-1 cell lines stably expressing negative control shRNA (sh-NC) and shAIM2. The results showed that IL-1β secretion (Revised Fig 4D) as well as IL-1β (p17) cleavage and Casp-1 (p20 and p22) maturation (Revised Fig 4E) induced by poly dA:dT were attenuated by sh-AIM2, however, IL-1β secretion (Revised Fig 4D) as well as IL-1β (p17) cleavage and Casp-1 (p20 and p22) maturation (Revised Fig 4E) induced by HSV-1 infection were unaffected by sh-AIM2.

**5.** STING recruits NLRP3 to the ER, but from Figure 5A, it shows that NLRP3 is partially locating in ER without STING’s recruitment.

In Figure 5B and 5C Calnexin and ER blue are performed to show the colocalization of ER and NLRP3, Zhijian Chen’s paper shows that NLRP3 aggregates in TGN under multiple stimulation such as Nigericin and ATP, could the authors stain TGN38/GM130 to determinate that NLRP3 is not locating in Golgi after HSV stimulation or HSV-120 transfection as well as stain EEA1 which could exclude the possibility that NLRP3 is not aggregated in endosome?

In Figure 5J, 5K and 5L, NLRP3 still significantly augment in ER after HSV stimulation under the condition of STING knock down, it is also important to use STING-/- cells to identify if STING deficiency could affect NLRP3’s translocation into ER.

**Authors’ Response:** Thank you for the comment.

- As you indicated, we have changed “recruit NLRP3 to the ER” to “facilitated NLRP3 ER localization” in the revised manuscript, as NLRP3 localized in ER without stimulation ([F1000Res.](https://www.ncbi.nlm.nih.gov/pubmed/?term=PMID%3A+31131091) 2019 May 17; 8: F1000 Faculty Rev-676. PMID: 31131091).

- We used Syntaxin 6 (TGN) and GM130 to demonstrated that NLRP3 is not locating in Golgi after HSV stimulation or HSV-120 transfection in the revised manuscript.

**-** We have changed “recruit NLRP3 to the ER” to “improved NLRP3 ER localization” in the revised manuscript. Even when we used the STING^-/-^ cells, we could also detect NLRP3 localization in ER.

**6.** In Figure 6, the author should explain why HA-UB (K48R) and HA-UB (K63R) constructs were used. In Figure 6B-C, the difference and function of K48R, K48O, K63R and K63O should be described in main text.

**Authors’ Response:** Thank you for your suggestions. We have described that post-translational modifications of NLRP3 are critical for its activation, including phosphorylation ([Front Immunol.](https://www.ncbi.nlm.nih.gov/pubmed/?term=43.%09Song+N%2C+Li+T.+2018.+Regulation+of+NLRP3+Inflammasome+by+Phosphorylation.+Front+Immunol+9%3A2305.) 2018 Oct 8;9:2305. PMID: 30349539), SUMOylation ([Nat Commun.](https://www.ncbi.nlm.nih.gov/pubmed/?term=Barry+R%2C+John+S+W%2C+Liccardi+G%2C+Tenev+T%2C+Jaco+I%2C+Chen+C+H%2C+Choi+J%2C+Kasperkiewicz+P%2C+et+al.+2018.+SUMO-mediated+regulation+of+NLRP3+modulates+inflammasome+activity.+Nat+Commun+9%3A3001.) 2018 Aug 1;9(1):3001. PMID: 30069026), and ubiquitination ([Mol Cell.](https://www.ncbi.nlm.nih.gov/pubmed/?term=Py+B+F%2C+Kim+M+S%2C+Vakifahmetoglu-Norberg+H%2C+Yuan+J.+2013.+Deubiquitination+of+NLRP3+by+BRCC3+critically+regulates+inflammasome+activity) 2013 Jan 24;49(2):331-8. PMID: 23246432) in the revision. The deubiquitination of NLRP3 has been reported to contribute to inflammasome activation ([Mol Cell.](https://www.ncbi.nlm.nih.gov/pubmed/?term=PMID%3A+23246432) 2013 Jan 24; 49(2): 331-8. PMID: 23246432; [EMBO J.](https://www.ncbi.nlm.nih.gov/pubmed/?term=PMID%3A+30787184) 2019 Mar 15; 38(6): e100376. PMID: 30787184). They focus on the K48- and K63- linked ubiquitination of NLRP3. Here, we have used the HA-UB (K48R) and HA-UB (K63R) constructs as well as K48O and K63O constructs, and have described the details of the four plasmids in the revised manuscript. K48R and K63R are one lysine residues mutant, while K48O and K63O are ubiquitin mutant that only retains a single lysine residue.

**7.** In Figure 8, the authors have used NLRP3-KO mice, however, STING-KO mice may be more important in this study to prove its essential role in NLRP3 inflammasome activation. In Figure 8C, a RNA virus, such as SeV or ZIKA, should be added as a control.

Authors’ Response: Thank you for your comments. STING-KO mice has been demonstrated to be essential for host defense against HSV-1 infection ([Nature](https://www.ncbi.nlm.nih.gov/pubmed/?term=PMID%3A+19776740) 2009 Oct 8; 461(7265): 788-92. PMID: 19776740). This is the first to demonstrate that NLRP3 deficiency mice are more susceptibility to HSV-1 infection and exhibit early onset of death upon the infection. Here we only wanted to further know the role of the STING-NLRP3 pathway in the regulation of HSV-1 infection.

**8.** In Figure 8, the author only described the important role of NLRP3 in host defense against viral infection. But whether the effect is related to the STING-NLRP3 association is not described. More experiments or description need to be added.

**Authors’ Response:** Thank you for your comments. We have demonstrated the important role of the STING-NLRP3 pathway in host defense upon HSV-1 infection in Fig. 2, 3, 4, 5, 6, and 7. First, STING binds to and improves NLRP3 localization in ER to promote the formation of the NLRP3 inflammasome. Second, STING interacts with NLRP3 and decreases K48- and K63-linked polyubiquitination of NLRP3 to induce the activation of the NLRP3 inflammasome. In the mouse model, we demonstrate that NLRP3 is essential for host defense against HSV-1 infection by facilitating IL-1β activation. Taken together, we reveal that the cGAS-STING-NLRP3 pathway plays key roles in the NLRP3 inflammasome activation and IL-1β secretion upon HSV-1 infection in the revised manuscript. Thus, we demonstrate that the link between the cGAS-STING-NLRP3 activation and HSV-1 infection is tight and clear.

**Reviewer #4:**

**1.** Based on Figure 1H and 1I, STING 1-160 (transmembrane domain) is responsible for the NLRP3 binding as well as NLRP3 function, can the authors show that STING 1-160 can recruit NLRP3 that same as STING WT, like in Figure 1L-M?

**Authors’ Response:** We have showed that like STING WT, STING 1-160 can also recruit NLRP3 in the revised manuscript.

**2.** Figure 5, upon HSV-1 infection as well DNA stimulation, STING has been reported to translocate to Golgi. Can the authors look further details on the location of STING regarding Golgi location? Moreover, in Figure 5H and 5I, it will be great to include STING IF staining.

**Authors’ Response:** As you suggested, we have used Syntaxin 6 (TGN) and GM130 to demonstrated that NLRP3 is not locating in Golgi after HSV stimulation or HSV-120 transfection in the revised manuscript. Our STING antibody isn’t suitable for IF staining.

**3.** Figure 8, it will be great if the authors can compare the IL-1b response in WT and STING-/- mice. (Do not have to do the survival curve, just show the IL-1b response in more than one organs like in 8 C, D, H, K)

**Authors’ Response:** It is good idea to compare the IL-1b response in WT and STING^-/-^ mice. However, we do not have the STING^-/-^ mouse model, and thus it is impossible for us to do the experiments. STING-KO mice has been demonstrated to be essential for host defense against HSV-1 infection ([Nature](https://www.ncbi.nlm.nih.gov/pubmed/?term=PMID%3A+19776740) 2009 Oct 8; 461(7265): 788-92. PMID: 19776740). The result is similarly to that obtained from our NLRP3 knockout mice. We believed that the STING-NLRP3 is essential for host defense against HSV-1 infection.

**Part III – Minor Issues: Editorial and Data Presentation Modifications**

Please use this section for editorial suggestions as well as relatively minor modifications of existing data that would enhance clarity.

**Reviewer #1:**

**Fig. 1C:** ASC and pro-CASP1 were expressed in lower level. Will it be expression problem so that only NLRP3 can be detected? Reciprocal IP on STING should be performed to address this problem.

**Authors’ Response:** As you suggested, the reciprocal IP on STING is performed in the revised manuscript.

**Fig. 1F,** lanes 5-8: It is difficult to conclude that LRR and NACHT were immunoprecipitated with STING. A better blot or labelling is required to demonstrate the corresponding bands of LRR and NACHT clearly. Secondary antibodies that react strongly with the heavy chain of IgG should be avoided.

**Authors’ Response:** Secondary antibodies that react strongly with the heavy chain of IgG is avoided in the revised manuscript.

**Fig. 1G, H and I**: Apparently TM4 of STING is also involved in the interaction with NLRP3. The pull down of NLRP3 was highly reduced in lane 7. A truncated mutant with only TM4 and TM5 removed (1-111, 160-379bp) should be constructed and its function on NLRP3 binding and activation should be verified. This should also be included in ubiquitination assays and other experiments.

**Authors’ Response:** TM5 deletion STING has constructed. The function of TM5 deletion STING on the NLRP3 binding and activation have verified, ubiquitination assays and other experiments have also included in the revised manuscript.

STING interacts with NLRP3 through TM5 domain (Fig. 1H) and loss of TM2 abolishes STING-mediated IL-1β induction (Fig 1I). Experiment presented in Fig. 1J, K should be repeated using STING binding-defective mutant (delTM5) and delTM3 to make the point that both TMs are necessary for STING-mediated NLRP3 activation. Truncated mutation of STING (delTM2 and delTM5) can also be used to strengthen the point that STING recruits and deubiquitinates NLRP3 upon HSV-1 infection (Fig. 5, 6).

**Authors’ Response:** STING binding-defective mutant (delTM5) has been used to demonstrate IL-1β induction, recruitment, and deubiquitinations of NLRP3 in the revised manuscript.

**Fig. 1J**: The label of input is missing in the lower panel.

**Authors’ Response:** As you suggested, we have added the label of input in the revised manuscript.

**Fig. 1K**: Oligomer was detected in negative control (ASC only).

**Authors’ Response:** In the revised manuscript, we showed that the oligomer of ASC was detected in the pellet of HEK293T cells overexpress ASC, NLRP3 expression enhanced the oligomer of ASC, and STING facilitated NLRP3-mediated ASC oligomerization. We also demonstrated that sh-STING significantly attenuated endogenous IL-1β secretion as well as IL-1β (p17) maturation and Casp-1 (p20 and p22) cleavage or ASC oligomerization induced upon HSV-1 infection, but had no effect on IL-1β secretion , IL-1β (p17) maturation and Casp-1 (p20 and p22) cleavage or ASC oligomerization induced by Nigericin stimulation in the revised manuscript (Revised Fig 7F, G, and I).

**Fig. 2B**: STING was also pulled down by negative control IgG.

**Authors’ Response:** We are sorry for the confusion. As correctly described in the revised Figure legends, cell lysates were subjected to Co-IP using anti-STING antibody. TPA-differentiated THP-1 macrophages were mock-infected or infected with HSV-1 (MOI=1) for 2 h and 4 h.

**Fig. 2G:** The IP label for the first lane should be Flag instead of IgG. STING can be pulled down in the first lane. The label should be similar to Fig. 2D.

Authors Answer: We have corrected the mistakes in the revised manuscript.

**Fig. 5:** Staining and fractionation assays were used to suggest that STING recruits NLRP3 to ER for inflammasome activation. NLRP3 and ASC speck formation should be analyzed to provide better evidence in support of the claim (Fig. 5H and 5I).

**Authors’ Response:** In the revised manuscript, we have demonstrated that sh-STING significantly attenuated endogenous IL-1β secretion as well as IL-1β (p17) maturation and Casp-1 (p20 and p22) cleavage or ASC oligomerization induced upon HSV-1 infection, but had no effect on IL-1β secretion , IL-1β (p17) maturation and Casp-1 (p20 and p22) cleavage or ASC oligomerization induced by Nigericin stimulation (Fig 7F, G, and I).

STING can be translocated into ERGIC and Golgi upon activation, to clarify whether NLRP3 is also recruited, Golgi and EGRIC markers should be used upon HSV-1 infection and HSV120-mer transfection. Moreover, it was previously reported that NLRP3 can be translocated to mitochondria through MAVS. Thus, NLRP3 should be costained with mitochondrial marker upon HSV-1 infection.

**Authors’ Response**: As you suggested, we have used Syntaxin 6 (TGN) and GM130 to demonstrated that NLRP3 is not locating in Golgi after HSV stimulation or HSV-120 transfection in the revised manuscript. The EGRIC antibody isn’t suitable for IF and we demonstrate that our fractionation assay just contained little EGRIC. We believed that STING improved NLRP3 ER localization and activated the inflammasome, but not into ERGIC and Golgi. STING can be translocated into ERGIC and Golgi upon HSV-1 infection, and thus we have detected the ERGIC and Golgi. However, the localization of NLRP3 is complicated ([F1000Res.](https://www.ncbi.nlm.nih.gov/pubmed/?term=PMID%3A+31131091) 2019 May 17; 8: F1000 Faculty Rev-676. PMID: 31131091).

For the fractionation assay (Fig. 5E, F, G, J, K, L), it is possible that the microsomal fraction might be contaminated by Golgi and EGRIC. Since it was not explained how fractionation was performed, Golgi and EGRIC markers should be analyzed by Western blotting.

**Authors’ Response:** We have mentioned that we used the Endoplasmic Reticulum Isolation Kit (ER0100) in the revised Material and Method. We also used the antibody to demonstrate that there is no Golgi and little EGRIC in the fraction in the revised manuscript.

In loss-of-function experiment, it was shown that knockdown of STING prevents NLRP3 translocation to ER (Fig. 5) and its deubiquitination (Fig. 6). The critical experiment here is the detection of IL-1β protein level (Fig. 7). Additional lines of results are required to address whether knockdown of STING reduces ASC speck formation or oligomerization upon HSV-1 infection. STING inhibitors can also be used to test this hypothesis.

**Authors’ Response:** We have demonstrated that sh-STING significantly attenuated endogenous IL-1β secretion as well as IL-1β (p17) maturation and Casp-1 (p20 and p22) cleavage or ASC oligomerization induced upon HSV-1 infection, but had no effect on IL-1β secretion , IL-1β (p17) maturation and Casp-1 (p20 and p22) cleavage or ASC oligomerization induced by Nigericin stimulation (Fig 7F, G, and I) in the revised manuscript. STING activation is complication, it may be the mechanism of the inhibitor is different to the pathway.

**Fig. 6:** Control of nigericin treated sample should be added to show the requirement of STING for HSV-1-induced NLRP3 inflammasome only.

**Authors’ Response:** We have demonstrated that sh-STING significantly attenuated endogenous IL-1β secretion as well as IL-1β (p17) maturation and Casp-1 (p20 and p22) cleavage or ASC oligomerization induced upon HSV-1 infection, but had no effect on IL-1β secretion , IL-1β (p17) maturation and Casp-1 (p20 and p22) cleavage or ASC oligomerization induced by Nigericin stimulation (Fig 7F, G, and I) in the revised manuscript.

**Fig. 8N, O:** H&E staining was shown for pathology of HSV-1-infected WT and NLRP3-/- mice. Tissue section of mock-infected WT and NLRP3-/- mice should also be shown.

**Authors’ Response**: As you suggested, tissue sections of mock-infected WT and NLRP3-/- mice were shown in the revised manuscript.

In text related to Fig. 8N and O. “Hematoxylin and Eosin (H&E) staining showed that more infiltrated neutrophils and mononuclear cells were detected in the lung and brain of infected WT mice as compared with NLRP3-/- mice (Fig 8N), and immunohistochemistry (IHC) analysis revealed that IL-1β protein level was higher in the lung and brain of infected WT mice as compared with NLRP3-/- mice (Fig 8O), revealing that NLRP3 deficiency mice are more susceptibility to HSV-1 infection and elicit weak inflammatory responses.” It should be mouse lung in Fig. 8N and mouse brain in Fig. 8O, not H&E in Fig. 8N and IHC in Fig. 8O.

**Authors’ Response:** We have corrected the mistakes in the revised manuscript.

**Reviewer #2: Minor:**

**1.** Introduction: Line 83-92: Very brief. Should cite the articles about HSV1 innate inflammasome and interferon responses and discuss the previous findings.

**Authors’ Response:** We have discussed the previous finding in the revised discussion section.

**2.** Transfection procedures are not given

**Authors’ Response:** We have indicated that we used the Lipofectamine 2000 that is purchased from Invitrogen Corporation (Carlsbad, CA, USA). The website has the detail transfection procedures.

**3.** Line 147-148. Give reference.

**Authors’ Response**: This is a well-known activator for NLRP3 inflammasome ([Nature](https://www.ncbi.nlm.nih.gov/pubmed/19339971) 2009 May 21; 459(7245): 433-6. PMID:19339971). We have added the reference in the revised manuscript.

**4.** Methodologies are not given for many experiments.

**Authors’ Response:** We have added some details in the revised manuscript.

**5.** Fig 9 legend- rewrite- line 874-876. Genomic DNA recognition by cGAS is not shown

**Authors’ Response:** It is well known that after the HSV-1 infection, the cGAS binds to DNA independently of its sequence; this binding activates cGAS to catalyze the synthesis of a unique cGAMP isomer, which contains both 2’–5’ and 3’–5’ phosphodiester linkages. This molecule, termed 2’3’cGAMP, functions as a second messenger that binds and activates the adaptor protein STING. STING then activates the protein kinases IKK and TBK1, which in turn activate the transcription factors NF-κB and IRF3 to induce interferons and cytokines in the revised manuscript ([Science](https://www.ncbi.nlm.nih.gov/pubmed/?term=PMID%3A+23989956) 2013 Sep 20; 341(6152): 1390-4. PMID: 23989956). We constructed THP-1 cell lines stably expressing negative control shRNA (sh-NC) and shRNAs (sh-IFI16 and cGAS). IL-1β secretion (Fig 4A) as well as IL-1β (p17) cleavage and Casp-1 (p20 and p22) maturation (Fig 4B) induced by HSV-1, not Nigericin, were attenuated by sh-IFI16 and sh-cGAS.

**Reviewer #3: Minor points:**

**1.** In this article, the author may need to show the pro-IL-1β and p17, pro-CASP-1 and p20 in one figure instead of cutting into pro- and mature forms.

**Authors’ Response:** We have detected pro-IL-1β and pro-CASP-1 in the cell lysates and secretion protein p17 or p20 in the supernatants, we could not show pro-IL-1β and p17, pro-CASP-1 and p20 in one figure.

**2.** Some references are inaccurate, for example, to describe “The NLRP3 inflammasome is required for HSV-1-induced IL-1β activation”, authors have cited an article “Herpes Simplex Virus 1 VP22 Inhibits AIM2-Dependent Inflammasome Activation to Enable Efficient Viral Replication [25]”.

**Authors’ Response:** We have corrected the mistakes in the revised manuscript.

**3.** In Figure 1A-C, there are two different controls (IgG or HA-vector) that the authors used in co-immunoprecipitation experiments. In consideration of over-expressed proteins rather than endogenous proteins in HEK293T cells, HA-vector is more suitable control.

**Authors’ Response:** We have used HA-vector in Fig 1A-C in the revised manuscript.

**4.** In line 270, L-1β should be IL-1β.

**Authors’ Response:** We have changed “L-1β” to “IL-1β” in the revised manuscript.

**Reviewer #4:**

**1.** MEF stands for “Mouse Embryonic Fibroblasts”, so “Mice” in front of “MEF” is redundant. Please note that there are multiple incidences in this manuscripts.

**Authors’ Response:** We have deleted the “Mice” in the revised manuscript.

**2.** I do not think sufficient data has been provided to conclude that STING “removes” K48- and K63-linked polyubiquitination of NLRP3, thereby promoting the inflammasome activation. There is also no published articles indicating that STING has DUB function. The authors instead showed that STING expression is correlated with the attenuation of both K48- and K63-linked polyubiquitination of NLRP3. Would the authors consider concluding the results in a different way?

**Authors’ Response:** STING has no DUB function. We think STING recruit another DUB for deubiquitination of NLRP3. However, we have not found such DUB protein and thus we have changed “removes polyubiquitination of NLRP3” to “decreases polyubiquitination of NLRP3” in the revised manuscript.

**3.** Line 353-364, would the authors please discuss possible mechanisms why STING regulates polyubiquitination of NLRP3? K63 linked polyubiquitination of NLRP3 has been shown to be critical for NLRP3 signaling. Would the authors discuss why removing this would benefit HSV-1 dependent NLRP3 activation?

**Authors’ Response:** We have described that post-translational modifications of NLRP3 are critical for its activation, including phosphorylation ([Front Immunol.](https://www.ncbi.nlm.nih.gov/pubmed/?term=43.%09Song+N%2C+Li+T.+2018.+Regulation+of+NLRP3+Inflammasome+by+Phosphorylation.+Front+Immunol+9%3A2305.) 2018 Oct 8;9:2305. PMID: 30349539), SUMOylation ([Nat Commun.](https://www.ncbi.nlm.nih.gov/pubmed/?term=Barry+R%2C+John+S+W%2C+Liccardi+G%2C+Tenev+T%2C+Jaco+I%2C+Chen+C+H%2C+Choi+J%2C+Kasperkiewicz+P%2C+et+al.+2018.+SUMO-mediated+regulation+of+NLRP3+modulates+inflammasome+activity.+Nat+Commun+9%3A3001.) 2018 Aug 1;9(1):3001. PMID: 30069026), and ubiquitination ([Mol Cell.](https://www.ncbi.nlm.nih.gov/pubmed/?term=Py+B+F%2C+Kim+M+S%2C+Vakifahmetoglu-Norberg+H%2C+Yuan+J.+2013.+Deubiquitination+of+NLRP3+by+BRCC3+critically+regulates+inflammasome+activity) 2013 Jan 24;49(2):331-8. PMID: 23246432) in the revision. The deubiquitination of NLRP3 has been reported to contribute to inflammasome activation ([Mol Cell.](https://www.ncbi.nlm.nih.gov/pubmed/?term=PMID%3A+23246432) 2013 Jan 24; 49(2): 331-8. PMID: 23246432; [EMBO J.](https://www.ncbi.nlm.nih.gov/pubmed/?term=PMID%3A+30787184) 2019 Mar 15; 38(6): e100376. PMID: 30787184). They focus on the K48- and K63- linked ubiquitination of NLRP3. Here, we have used the HA-UB (K48R) and HA-UB (K63R) constructs as well as K48O and K63O constructs, and have described the details of the four plasmids in the revised manuscript. K48R and K63R are one lysine residues mutant, while K48O and K63O are ubiquitin mutant that only retains a single lysine residue.
