# Supplementary material for: STING promotes NLRP3 localization in ER and facilitates NLRP3 deubiquitination to activate the inflammasome upon HSV-1 infection
Source: PLoS Pathog. 2020 Mar 18;16(3):e1008335. doi: 10.1371/journal.ppat.1008335 (PMC7080238; doi:10.1371/journal.ppat.1008335)
Supplement: Attachment — Submitted filename: PLoS Pathog Responses to the Reviews 20200115.docx [file ppat.1008335.s004.docx]

**Responses to the Reviews**

**Part I - Summary**

Please use this section to discuss strengths/weaknesses of study, novelty/significance, general execution and scholarship.

Reviewer #1: Most concerns raised have been addressed.

**Authors’ Response:** Thank you.

Reviewer #3: This manuscript has been greatly improved, and I am satisfied for the responses.

**Authors’ Response:** Thank you very much for the comment.

Reviewer #4: The authors made significant progress and adequately addressed my comments scientifically by either experiments or discussion. I would recommend acceptance. Well done and look forward to the authors' future work.

**Authors’ Response:** Thank you very much for the comments and for your recommendation.

**Part II – Major Issues: Key Experiments Required for Acceptance**

Please use this section to detail the key new experiments or modifications of existing experiments that should be absolutely required to validate study conclusions.

Generally, there should be no more than 3 such required experiments or major modifications for a "Major Revision" recommendation. If more than 3 experiments are necessary to validate the study conclusions, then you are encouraged to recommend "Reject".

Reviewer #1: Nil.

**Authors’ Response:** Thank you.

Reviewer #3: No more experiment is required.

**Authors’ Response:** Thank you.

Reviewer #4: The authors made significant progress in the last revision and adequately addressed my comments scientifically by either experiments or discussion.

**Authors’ Response:** Thank you very much for the comments.

**Part III – Minor Issues: Editorial and Data Presentation Modifications**

Please use this section for editorial suggestions as well as relatively minor modifications of existing data that would enhance clarity.

Reviewer #1: 1) The pixel and resolution of some Western blots are low, e.g. Fig. 3C and D Sup, Fig. 6F and I and Fig. 7G. Higher resolution blots should be provided for publication.

**Authors’ Response:** Thank you very much for the comments.

According to your suggestions, we have improved the pixel and resolution of Western blots in most figures in the revisions. We are sure higher resolution blots will be provided for publication.

In addition, densitometry should be performed for some of the blots if comparison is made, e.g. Fig. 2F, 3C, 3D, 3J, 4B, 4E, 4I, 4J, 5F-H, 5K-M, 7B, 7E, 7G and 7K.

**Authors’ Response:** Thank you for the comments.

As you suggested, we have performed densitometry by Image J for the blots in Fig. 2C, 2D, 2F, 2G, 5F, 5G, 5H, 5K, 5L, and 5M in the revised manuscript.

2) For all confocal images, it would be desirable to show more than one cell (Fig. 1N, 5A, 5B, 5D, 5I, 5J).

**Authors’ Response:** Thank you for the comments.

According to your suggestions, we have showed more than one cell in Fig. 1N, 5A, 5B, 5D, 5I, and 5J in the revised manuscript.

**Reviewer #3: (No Response)**

**Authors’ Response:** Thank you.

Reviewer #4: The authors made significant progress in the last revision and adequately addressed my comments scientifically by either experiments or discussion.

**Authors’ Response:** Thank you very much.
